# Identification of predictive biomarker signatures in melanoma tumors associated with response to tumor-infiltrating lymphocyte (TIL) therapy

**DOI:** 10.1186/2051-1426-1-S1-P48

**Published:** 2013-11-07

**Authors:** Jie Qing Chen, Caitlin Creasy, Carlos Antonio Torres Cabala, Suhendan Ekmekcioglu, Sourindra N Maiti, Jason Roszik, Cara Haymaker, Chantale Bernatchez, Patrick Hwu, Laszlo Radvanyi

**Affiliations:** 1 UT MD Anderson Cancer Center, Houston, TX, USA

## 

Adoptive cell therapy using expanded autologous TIL is a promising immunotherapy for metastatic melanoma. However, we still know relatively little about the types of T cells in TIL mediating tumor regression, and there have been no biomarker studies on the actual tumors used to expand TIL to identify factors predictive of clinical response. The purpose of this study was to identify immune biomarkers in tumors used to expand TIL and determine their power to predict an eventual response to TIL therapy. We performed immunohistochemistry (IHC) for a number of parameters (e.g., CD8, CD4, CD3, FoxP3, and PD-1) on formalin-fixed paraffin-embedded (FFPE) tumor samples from 48 patients that expanded TIL and were eventually treated as well as FFPE samples from 39 patients from which TIL did not successfully expand. We found a correlation between the percentage of CD8+ cells in the original tumor by IHC and the CD8 content of the final expanded TIL product of these patients (p=0.046, r2=0.283, Spearman correlation coefficient). We also found highly significant differences in CD8, CD4 and CD3 staining in tumors between TIL growers and non-growers (p<0.0001). Although the percentages of total, peri-tumoral or intra-tumoral CD8, PD-1 and FoxP3 in the original tumor for TIL expansion could not predict tumor response (CR/PR vs. SD/PD), increased peri-tumoral and total CD4 staining had an inverse trend towards predicting CR/PR (p=0.067 and p=0.082, respectively). Interestingly, when we combined CD8 and FoxP3 staining and developed a scoring system that considered the location and extent of staining, we found that patients who had a lower CD8 and FoxP3 score in peri-tumoral and/or intra-tumoral areas had significantly shorter overall survival in both univariate (HR= 3.657, p=0.041) and multivariate (HR=3.514, p=0.048) Cox proportional hazards analysis. We also performed gene expression analysis on RNA isolated from the same tumor samples using a new NanoString nCounter™ technology. This revealed significant differences in a number of immune system-related genes in tumors of TIL growers and non-growers, such as CD3ε and CD3δ, STAT4, TRAFs, CD27, ICOS, IL-21R, TNF-α, FoxP3, and CCR8. There were also differences in gene expression in tumors of responding and non-responding patients treated with TIL, such as BATF3 and IRAK1. Our data suggests that IHC analysis together with gene profiling of archived FFPE tumor samples is useful to develop predictive biomarker signatures to select patients for TIL therapy and identify those who will have a survival benefit for stratification in future clinical trials.

